# Clinical Efficacy of 10 Min of Active Prewarming for Preserving Patient Body Temperature during Percutaneous Nephrolithotomy: A Prospective Randomized Controlled Trial

**DOI:** 10.3390/jcm13071843

**Published:** 2024-03-22

**Authors:** Jung-Woo Shim, Hyejin Kwon, Hyong Woo Moon, Min Suk Chae

**Affiliations:** 1 Department of Anesthesiology and Pain Medicine, Seoul St. Mary’s Hospital, College of Medicine, The Catholic University of Korea, Seoul 06591, Republic of Korea; 2 Department of Anesthesiology and Pain Medicine, Yeouido St. Mary’s Hospital, College of Medicine, The Catholic University of Korea, Seoul 06591, Republic of Korea; 3 Department of Urology, Seoul St. Mary’s Hospital, College of Medicine, The Catholic University of Korea, Seoul 06591, Republic of Korea

**Keywords:** prewarming, hypothermia, body temperature, percutaneous nephrolithotomy

## Abstract

**Background:** Percutaneous nephrolithotomy (PNL) poses a risk of hypothermia. Additionally, general anesthesia lowers the thresholds for shivering and vasoconstriction, which leads to dysfunction of central thermoregulation. Perioperative hypothermia is associated with adverse outcomes after surgery. In this study, we aimed to demonstrate that prewarming for 10 min can effectively prevent early hypothermia during PNL. **Methods:** A total of 68 patients scheduled for elective PNL were recruited to this study from January to June 2022, but two patients were excluded because of a change in the surgical plan. After randomization, patients in the prewarming group (*n* = 32) received warming using a forced-air warming device for 10 min in the preoperative area before being transferred to the operating room, while the controls (*n* = 34) did not. The incidence of hypothermia within the first hour after inducing general anesthesia was the primary outcome. Perioperative body temperatures and postoperative recovery findings were also evaluated. **Results:** Early intraoperative hypothermia decreased significantly more in the prewarming group than in the control group (9.4% vs. 41.2%, *p* = 0.003). Moreover, the net decrease in core body temperature during surgery was smaller in the prewarming group than in the control group (0.2 °C, vs. 0.5 °C, *p* = 0.003). In addition, the prewarmed patients had a lower incidence of postoperative shivering and a shorter post-anesthesia-care unit (PACU) stay (12.5% vs. 35.3%, *p* = 0.031; and 46 vs. 50 min, *p* = 0.038, respectively). **Conclusions:** Prewarming for 10 min decreased early hypothermia, preserved intraoperative body temperature, and improved postoperative recovery in the PACU.

## 1. Introduction

Thermoregulation in humans encompasses mechanisms such as sweating, shivering, vasodilation, and vasoconstriction [1]. However, general anesthesia disrupts this balance by lowering the thresholds for shivering and vasoconstriction, thereby impairing the body’s central thermoregulatory functions [2]. Particularly noteworthy is the first hour post-anesthesia induction, during which the redistribution of heat from the core to the peripheral parts of the body leads to a sharp decline in core body temperature. The extent of this temperature drop is influenced by the temperature differential between the body’s central and peripheral regions [3]. Perioperative hypothermia, a consequence of this thermal imbalance, is linked to various negative outcomes, such as extended hospital stays and an increased incidence of postoperative shivering [4]. Even a slight drop in intraoperative body temperature can lead to higher patient care costs [5]. Conversely, maintaining normothermia during surgical procedures can reduce oxygen consumption, lower the risk of wound infections, minimize bleeding, and alleviate pain [6]. Thus, the use of active warming techniques is recommended over passive methods to promote better post-surgery recovery. Prewarming patients—increasing the body’s surface temperature before anesthesia induction—can mitigate the core-to-peripheral heat redistribution by reducing the initial temperature gradient, thereby decreasing the potential for hypothermia [2,7].

Percutaneous nephrolithotomy (PNL), a critical surgery for large urinary stones, relies heavily on fluoroscopy or ultrasonography to guide it through the kidney’s intricate calyceal system [8,9]. The extensive use of irrigation fluids, essential for maintaining a clear surgical view, unfortunately leads to a high incidence of hypothermia among patients, exacerbated by the room temperature of these fluids [10,11,12]. The situation is further complicated by the surgery’s duration, the cool operating room environment, and the difficulty in applying effective warming methods due to the procedural location [10,12,13]. While forced-air warming is a common strategy to counteract hypothermia, its efficiency is notably diminished in PNL due to the prone positioning of patients, limiting the body surface area available for warming [14,15,16]. The National Institute for Health and Clinical Excellence (NICE) has recommended a 30 min prewarming period to mitigate this risk, but the practicality of such a recommendation in a busy surgical schedule is questionable [17]. Emerging research suggests that even a shortened prewarming phase could be beneficial, highlighting the need for further studies to explore the balance between clinical effectiveness and operational feasibility in the prevention of hypothermia during PNL procedures [7,18]. 

In this study, we aimed to demonstrate the efficacy of prewarming for 10 min in preventing early intraoperative hypothermia during PNL. We hypothesized that prewarming preserves intraoperative body heat in patients undergoing PNL and can thus overcome the limitations of intraoperative warming methods.

## 2. Patients and Methods

### 2.1. Compliance with Ethical Standards

This prospective randomized trial was approved on 9 February 2021 by the Institutional Review Board and Ethics Committee of Seoul St. Mary’s Hospital, Catholic University of Korea, a tertiary academic teaching facility (approval number: KC21EISI0010). This study protocol was prospectively registered on a publicly accessible site, recognized by the International Committee of Medical Journal Editors (Clinical Research Information Service, Republic of Korea, approval number: KCT0005969). The principles of the Declaration of Helsinki were followed, and written informed consent was obtained from all participants.

### 2.2. Exclusion and Inclusion Criteria 

Patients scheduled for elective PNL were recruited for this study the day before surgery from January to June 2022. The exclusion criteria were age < 19 years or ≥80 years; American Society of Anesthesiologists Physical Status score ≥ 3; refusal to participate in the study; emergency case; severe intraoperative hemodynamic instability requiring continuous vasopressor infusion; and a change in the intraoperative surgical plan from PNL to ureteroscopic lithotripsy.

### 2.3. Randomization

Eligible patients were randomly divided into two groups using the sealed envelope method, i.e., control and prewarming groups. An independent colleague randomly divided the envelopes into groups of 10 with a 1:1 ratio to produce an equal distribution during the study period. The envelopes were stacked and stored. The topmost envelope was opened immediately after the patient arrived in the preoperative holding area (PHA) by a physician who was responsible for prewarming and was not involved in further patient care or data collection.

### 2.4. Active Prewarming Regimen in the Preoperative Holding Area

The patients in the prewarming group were warmed for 10 min upon arrival at the PHA. Active warming, using a forced-air warmer (Bair Hugger Warming Unit, Model 77500; 3M Healthcare, Eden Prairie, MN, USA), was performed with a cotton blanket covering the entire body of the patient while they lay on a bed. The temperature output of the warmer was 43 °C. All control group patients were covered with a cotton blanket over their entire body, but forced-air warming was not used. All patients were sent to the operating room after a 10 min stay in the PHA.

### 2.5. Perioperative Body Temperature Measurements

Upon arrival at the PHA, and before anesthesia was induced in the operating room, body temperature was measured with an infrared tympanic thermometer (ThermoScan; B. Braun, Berlin, Germany). After anesthesia was induced, intraoperative core body temperature was measured using an esophageal temperature probe until the end of anesthesia. During the PACU stay, body temperature was measured with an infrared tympanic thermometer.

### 2.6. Anesthetic and Surgical Management in the Operating Theater

Propofol (1.5–2 mg/kg) and rocuronium (0.8–1 mg/kg) were administered to induce anesthesia in the operating room. After loss of consciousness and self-breathing, an endotracheal tube and esophageal temperature probe were inserted (Top Probe; Top Medics, Seoul, Republic of Korea). The esophageal temperature probe was inserted to a depth equal to the length from the incisor to the xiphoid process, locating the probe tip at the lower thoracic vertebral level [19]. To maintain adequate depth of anesthesia and analgesia, inhaled desflurane (4–6%) and intravenous remifentanil (0.02–0.10 µg/kg/min) were administered for the remainder of the surgery. The target bispectral index was 40–60. Room temperature intravenous fluids were infused according to the calculated maintenance doses.

A balloon occlusion catheter was placed at the beginning of surgery by an experienced urologist under cystoscopic guidance in the frog-leg position. Then, a percutaneous approach was followed under fluoroscopic guidance in the prone position. Room temperature saline was used for irrigation, and the ambient temperature of the operating room was 23.0 °C (controlled by a central thermostat). All patients received forced-air warming using a forced-air cotton blanket positioned above the operative site in the prone position. The temperature output of the warmer was 38 °C during surgery.

Patients were placed in the supine positioned after completion of the surgery. After desflurane and remifentanil were discontinued, sugammadex (4 mg/kg) was injected to reverse the neuromuscular blockade. Patients were sent to the post-anesthetic care unit (PACU) after extubation.

After arriving in the PACU, all patients were covered with a cotton blanket. Full-body active warming was performed using a forced-air warming device with a temperature output of 43 °C if a patient was shivering or had a body temperature <36.0 °C. Pethidine (25 mg) was administered for continuous postoperative shivering despite the use of active warming. After a 30 min stay in the PACU and the achievement of a post-anesthesia recovery score ≥ 9, patients were sent to the general ward [20].

### 2.7. Primary and Secondary Outcomes

The primary outcome of the study was the incidence of early intraoperative hypothermia. Current guidelines define hypothermia as a core body temperature < 36.0 °C [21]. We considered any occurrence of hypothermia within the first hour of anesthesia as early intraoperative hypothermia. The secondary outcomes of the study were the degree of change in body temperature during anesthesia and the incidence of postoperative shivering. Shivering was defined as fasciculation involving the head, neck, or shoulders, or generalized hyperactivity [22]. 

### 2.8. Clinical Variables

The demographic findings included sex, age, body mass index, ASA classification I and II, and comorbidities (i.e., hypertension, diabetes mellitus, hepatitis B, and tuberculosis). The surgical findings included operation site (right, left, or both sides), total case length, patient positioning time, and administered volumes of irrigation and intravenous fluids. Duration of recovery in the PACU, hospital stay, and Clavien–Dindo classification were also recorded.

### 2.9. Sample Size and Statistical Analysis 

A retrospective chart review was performed to investigate the incidence of early intraoperative hypothermia in patients undergoing PNL at our institute. The review revealed that 50% of the patients who did not receive prewarming developed early intraoperative hypothermia. A reduction of two-thirds in the incidence of early intraoperative hypothermia was considered clinically meaningful. Thus, it was assumed that 16.5% of prewarmed patients would experience early intraoperative hypothermia. We calculated the sample size using a two-sided Fisher’s exact test at a significance level of *p* < 0.05. With a 5% risk of type 1 error and a 20% risk of type 2 error, 30 patients were required in each group. We enrolled 68 patients, considering a dropout rate of 10%.

The normality of the continuous data was assessed using the Shapiro–Wilk test. Descriptive statistics for categorical variables are provided as numbers (%), and continuous variables are provided as means (standard deviations) or medians (interquartile ranges), according to the normality of the variables. The *χ*^2^ or Fisher’s exact test was performed to compare categorical variables, as appropriate. Student’s *t*-test or the Mann–Whitney *U* test was used to compare continuous variables, as appropriate. Serial changes in perioperative core body temperature were evaluated using the Friedman test, with post hoc analysis performed using the Wilcoxon signed–rank test. All statistical tests were two-sided, and a *p*-value < 0.05 was considered significant. All analyses were performed with SPSS Statistics software (version 24.0; IBM Corp., Armonk, NY, USA).

## 3. Results

### 3.1. Comparison of Demographic and Surgical Findings between the Prewarming and Control Groups

**Figure 1:**
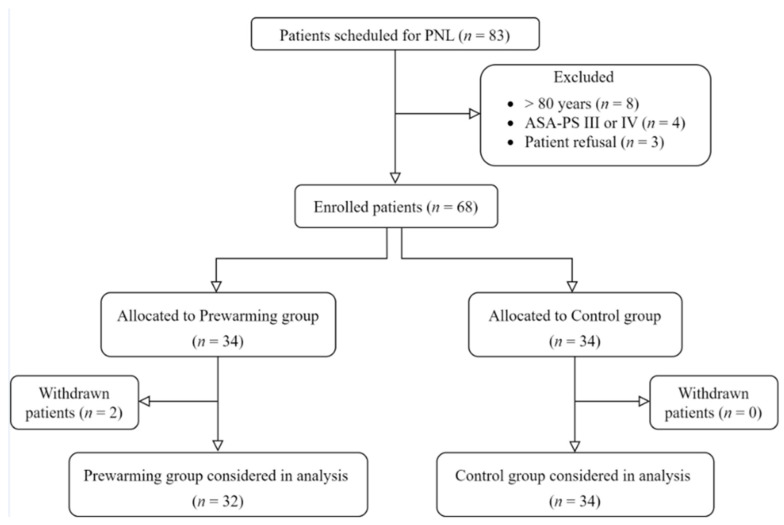
Flow diagram of the study. PNL, percutaneous nephrolithotomy; ASA-PS, American Society of Anesthesiologists Physical Status.

During the study period, 68 patients were enrolled in this study after excluding 15 who met the exclusion criteria; eight patients were aged >80 years, four had an American Society of Anesthesiologists Physical Status score of 3, and three refused to participate. In addition, two patients in the prewarming group underwent ureteroscopic lithotripsy because of a change in their surgical plan and were excluded from the study. Finally, 32 patients in the prewarming group and 34 patients in the control group completed the study (Figure 1).

No differences in demographic or surgical features were detected between the two groups (Table 1).

### 3.2. Comparison of Perioperative Body Temperature Findings between the Prewarming and Control Groups 

**Figure 2:**
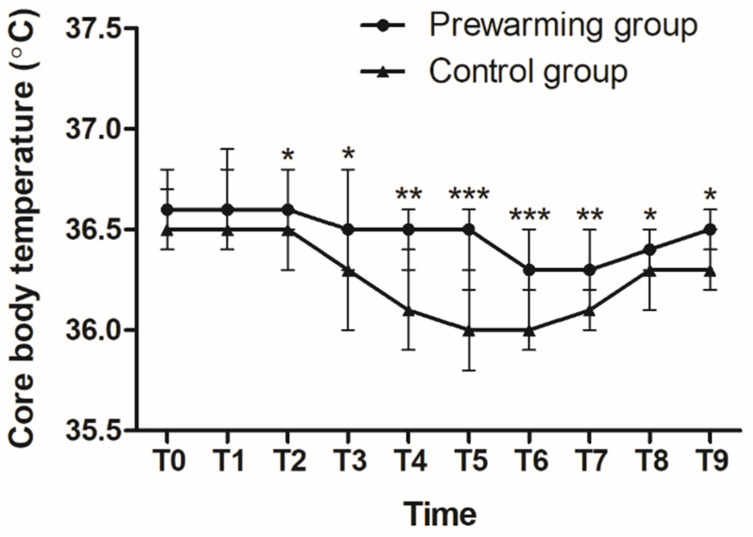
Serial changes in perioperative core body temperatures. Core body temperature was recorded upon arrival in the preoperative holding area (T0) before anesthesia was induced (T1), 15 min after anesthesia was induced (T2), 30 min after anesthesia was induced (T3), 45 min after anesthesia was induced (T4), 60 min after anesthesia was induced (T5), at the end of anesthesia (T6), upon arrival in the PACU (T7), 15 min after arrival in the PACU (T8), and upon discharge from the PACU (T9). * *p*-value of <0.05, ** *p*-value of <0.01, and *** *p*-value of <0.001. PACU, post-anesthetic care unit.

The frequency of early intraoperative hypothermia decreased more significantly in the prewarming group than in the control group (9.4% vs. 41.2%, *p* = 0.003, Table 2). As shown in Figure 2, the core body temperatures upon arrival in the PHA were comparable between the two groups. The 10 min prewarming did not result in a group difference in body temperature until anesthesia was induced. No significant increase in core body temperature was observed upon the induction of anesthesia, even in the prewarmed patients, compared to the measurements obtained in the PHA (*p* = 0.246).

However, core body temperature was significantly higher in the prewarming group than in the control group during general anesthesia (36.6 vs. 36.5 °C, *p* = 0.022, 15 min after anesthesia was induced; 36.5 vs. 36.3 °C, *p* = 0.015, 30 min after anesthesia; 36.5 vs. 36.1 °C, *p* = 0.002, 45 min after anesthesia; 36.5 vs. 36.0 °C, *p* < 0.001, 60 min after anesthesia; and 36.3 vs. 36.0 °C, *p* < 0.001, at the end of anesthesia, respectively). Moreover, the net decrease in core body temperature during surgery was significantly smaller in the prewarming group than in the control group (0.2 vs. 0.5 °C, *p* = 0.003). The two groups also differed in body temperature during the PACU stay (36.3 vs. 36.1 °C, *p* = 0.001, upon arrival in the PACU; 36.4 vs. 36.3 °C, *p* = 0.02, 15 min after arrival in the PACU; and 36.5 vs. 36.3 °C, *p* = 0.02, and upon discharge from the PACU, respectively). 

### 3.3. Comparison of Postoperative Recovery Findings between the Prewarming and Control Groups

The prewarmed patients had a lower incidence of postoperative shivering (12.5% vs. 35.3%, *p* = 0.031, Table 3). In addition, recovery time in the PACU was shorter in the prewarmed patients than in the control group (46 vs. 50 min, *p* = 0.038).

However, no significant differences in the duration of hospital stay and the incidence of perioperative complications were observed between the groups. Two patients in the prewarming group underwent reoperation surgery to remove residual urinary stones, and one patient in the control group suffered from bowel injury during surgery. However, the other patients were discharged without significant complications.

## 4. Discussion

The main finding of this study was that 10 min of preoperative warming using a forced-air warming device was effective in reducing early hypothermia during PNL. In addition, the prewarmed patients experienced less of a decrease in their core body temperature during surgery, compared to the controls. Prewarming also significantly decreased the incidence of shivering and recovery duration in the PACU.

Earlier studies demonstrated that short-term prewarming does not significantly increase initial core body temperature [7,18]. However, it effectively increases peripheral temperatures, thereby reducing the gradient between core and peripheral body temperatures. This reduction in gradient diminishes the extent of thermal redistribution during surgery, ultimately leading to better maintenance of core body temperature throughout the procedure [23]. A notable study on liver transplantation by Oh et al. highlighted that the risk of intraoperative hypothermia was considerably lower in the prewarming group compared to the non-prewarming group (60.0% vs. 95.0%, *p* = 0.02). This difference was especially pronounced immediately after anesthesia induction, with hypothermia incidence being significantly lower in the prewarming group during the post-induction phase (20.0% vs. 85.0%, *p* < 0.001) compared to later surgical phases. This suggests that prewarming primarily counteracts the post-induction redistribution of heat from the core to the periphery. Additionally, the duration of hypothermia was significantly shorter in the prewarming group (60 [0–221] minutes vs. 383 [108–426] minutes, *p* = 0.001). These findings demonstrate that prewarming practices, including the use of forced-air warming, can be beneficial in maintaining intraoperative body temperature during complex and lengthy surgeries like liver transplantation [24]. The increase in total body heat content before anesthesia and the reduction in core temperature drop immediately after anesthesia induction underline the importance of minimizing core-to-peripheral heat redistribution [18]. This supports our study’s results, showing that a 10 min prewarming period effectively controls intraoperative core body temperature in a similar manner.

In research involving volunteers under general anesthesia, it was discovered that forced-air prewarming for more than 30 min effectively increased peripheral tissue heat content [25]. For patients undergoing major surgeries, a prewarming duration of 60 min was adequate to prevent postoperative hypothermia [26]. However, these time frames may not be feasible in everyday clinical practice. Horn et al. found that patients who were not prewarmed experienced a greater decrease in core temperature compared to those who were prewarmed, even with active warming during surgery. Interestingly, even a brief 10 min prewarming period was effective in preventing hypothermia. Extending prewarming to 20 or 30 min did not significantly alter core temperature or reduce the rate of postoperative hypothermia much further (from 13% to 7% and 6%, respectively) [18]. Forbes et al. recommended employing both forced-air and intravenous fluid warming systems in conjunction to keep patients warm, in addition to keeping the operating room temperature at 22 °C, particularly for surgical procedures anticipated to exceed 30 min in duration [21]. However, the uptake of these recommendations was limited, partly because early research highlighted the benefits of 60–120 min of prewarming, which was deemed impractical for routine application [27,28,29]. Sessler et al. conducted a controlled study with volunteers to determine the optimal duration for effective prewarming. They concluded that 30 min of forced-air warming significantly increased tissue heat content, more so than previous studies suggested [3,25]. However, half of the participants reported uncomfortable sweating after one hour of prewarming, leading to a recommendation for a prewarming duration of between 30 and 60 min [25]. Bräuer et al. proposed that even shorter prewarming periods of less than 30 min might be adequate [15]. Given the efficiency of modern warming systems in covering a broad skin area and transferring heat to tissues effectively, 10 min prewarming could be a practical option for patients undergoing procedures like PNL. While our study did not directly compare the effects of 10 min versus 30 min prewarming, the findings suggest that a 10 min approach could suffice for maintaining heat in such patients.

Numerous studies have indicated that the use of warm irrigation fluids during PNL significantly enhances patients’ thermal comfort [11,12]. A notable study by Hosseini et al. found that while the initial core temperatures of patients were similar across different groups, all 20 patients in the group receiving cold irrigation fluids experienced hypothermia (*p* = 0.012). There was a marked difference in the final temperatures and the extent of temperature change between groups, with the warm fluid group showing higher final temperatures and lesser temperature variations compared to those receiving room temperature or cold fluids (*p* = 0.001). Additionally, the rate of shivering was lower in the warm solution group, although the difference was not statistically significant (*p* = 0.198) [11]. However, the utilization of warm irrigation fluids carries the risk of fluid overload due to a decrease in dynamic viscosity at elevated temperatures, which affects both density and fluidity. Specifically, increasing the temperature of the irrigation solution from 17 °C to 37 °C was predicted to raise the mean absorption rate by approximately 54%, consequently reducing the theoretical ‘safe’ duration of surgery by 65% for both electrolyte and non-electrolyte solutions. This reduction translates to an average decrease of 21.1 min for non-electrolyte solutions (from 60.0 to 38.9 min) and 35.2 min for electrolyte solutions (from 100.0 to 64.8 min), highlighting a significant risk of fluid overload with isothermic solutions due to the lower dynamic viscosity at higher temperatures [30]. While the administration of warm intravenous fluids has been shown to prevent perioperative hypothermia, its effectiveness is contingent on the volume and rate of fluid delivery [31]. An active prewarming strategy may offer an alternative means of preventing hypothermia during PNL without the need to warm the large volumes of irrigation fluids used in the procedure, especially considering that patients undergoing PNL receive intravenous fluids based on their basal fluid requirements. This approach suggests a potential strategy for maintaining normothermia in PNL patients while mitigating the risks associated with warm irrigation fluids [12]. 

Our study evaluated the effect of prewarming in the PHA for preventing early intraoperative hypothermia within the first hour of anesthesia. Core-to-peripheral redistribution of body heat occurs during the first hour after induction of anesthesia, and the core temperature begins to rise after thermal redistribution has occurred [32]. Thus, preventing early hypothermia was thought to be more important than preventing hypothermia during the remainder of the surgery. In addition, although prewarming was performed in the PHA, the transfer time to the operating room had no adverse impact on the maintenance of the effect of active warming [18]. Prewarming also significantly improved postoperative shivering. A program for enhanced recovery after surgery emphasizes the importance of preventing perioperative shivering, which delays patient recovery by increasing oxygen consumption, catecholamine release, infection rates, and the risks of hypoxemia or lactic acidosis. Thus, the preventive role of prewarming on shivering in the PACU has clinical significance [33,34].

The present study had some limitations. First, the study patients were aware of the groups they were allocated to, although a randomized assignment was used. The medical staff, nurses in charge of surgery and postoperative care, and the researchers were blinded to the group allocations. Second, we used a tympanic thermometer in conscious patients and an esophageal temperature probe in anesthetized patients. The utilization of less invasive temperature measurements was necessary for conscious patients, although accuracy may have been affected. Lastly, generalizing the effects of 10 min of prewarming to other surgeries is difficult. This study was performed in patients undergoing PNL, which usually takes 1–2 h. The minimum duration of prewarming required for preventing intraoperative hypothermia is 10–30 min depending on clinical conditions [7,18,35,36]. Further studies across diverse major surgical interventions are crucial for overcoming these existing limitations and extending the relevance of outcomes. Examining various prewarming intervals, such as 5, 10, 20, and 30 min, will deepen insights into clinical significance and potentially establish suitable minimum prewarming durations beneficial for patients with individual conditions.

## 5. Conclusions

Prewarming for 10 min significantly reduced early intraoperative hypothermia in patients undergoing PNL. The incidence of shivering and the recovery duration after surgery decreased significantly in the prewarmed patients. Future studies are needed to establish the efficacy of short-term prewarming.

## Figures and Tables

**Table 1:** Patients’ demographic and surgical findings.

Group	Prewarming Group	Control Group	*p* Value
	*n* = 32	*n* = 34	
***Demographic findings***			
Sex (male)	25 (78.1%)	23 (63.7%)	0.339
Age (years)	63 (52–71)	62 (50–68)	0.369
Body mass index (kg/m^2^)	23.8 (22.2–25.1)	23.3 (22.4–26.1)	0.812
ASA classification			0.601
I	5 (15.6%)	7 (20.6%)	
Ⅱ	27 (84.4%)	27 (79.4%)	
Comorbidity			
Hypertension	10 (31.3%)	8 (23.5%)	0.482
Diabetes mellitus	17 (51.3%)	15 (44.1%)	0.464
Hepatitis B	2 (6.3%)	2 (5.9%)	1.000
Tuberculosis	2 (6.3%)	4 (11.8%)	0.673
***Surgical findings***			
Operation site			0.194
Right side	11 (34.4%)	16 (47.1%)	
Left side	19 (59.4%)	13 (38.2%)	
Both side	2 (6.3%)	5 (14.7%)	
Total case length (min)	90 (72–109)	93 (70–116)	0.753
^†^ Patient positioning time (min)	13 (12–17)	12 (12–17)	0.285
Irrigation fluids (mL)	8550 (7300–10,175)	8200 (7075–11,375)	0.918
Intravenous fluids (mL)	200 (135–300)	200 (200–300)	0.487

Data are presented as median (25–75% interquartile range) or *n* (%). ^†^ Patient positioning time was defined as the time between the induction of anesthesia and the start of surgery after prone positioning; ASA, American society of anesthesiologists.

**Table 2:** Core body temperatures at each assessment and incidence of hypothermia.

Group	Prewarming Group	Control Group	*p* Value
	*n* = 32	*n* = 34	
***In the preoperative and intraoperative period***			
Core body temperature at arrival in the preoperative holding area (°C) (T0)	36.6 (36.4–36.8)	36.5 (36.4–36.7)	0.307
Core body temperature before the induction of anesthesia (°C) (T1)	36.6 (36.5–36.9)	36.5 (36.4–36.8)	0.151
Core body temperature 15 min after anesthesia (°C) (T2)	36.6 (36.5–36.8)	36.5 (36.3–36.6)	0.022
Core body temperature 30 min after anesthesia (°C) (T3)	36.5 (36.3–36.8)	36.3 (36.0–36.5)	0.015
Core body temperature 45 min after anesthesia (°C) (T4)	36.5 (36.3–36.6)	36.1 (35.9–36.4)	0.002
Core body temperature 60 min after anesthesia (°C) (T5)	36.5 (36.2–36.6)	36.0 (35.8–36.3)	<0.001
Core body temperature at the end of anesthesia (°C) (T6)	36.3 (36.2–36.5)	36.0 (35.9–36.2)	<0.001
^‡^ Early intraoperative hypothermia	3 (9.4%)	14 (41.2%)	0.003
Net decrease in core body temperature during surgery	0.2 (0.1–0.4)	0.5 (0.3–0.6)	0.003
***In the postoperative period***			
Core body temperature at arrival in the PACU (°C) (T7)	36.3 (36.2–36.5)	36.1 (36.0–36.3)	0.001
Core body temperature 15 min after the PACU arrival (°C) (T8)	36.4 (36.3–36.5)	36.3 (36.1–36.4)	0.020
Core body temperature at discharge from the PACU (°C) (T9)	36.5 (36.4–36.6)	36.3 (36.2–36.5)	0.020

Data are presented as median (25–75% interquartile range) or *n* (%). ^‡^ Early intraoperative hypothermia was defined as the occurrence of core body temperature below 36 °C during the first hour of surgery; PACU, post-anesthetic care unit.

**Table 3:** Postoperative recovery findings.

Group	Prewarming Group	Control Group	*p* Value
	*n* = 32	*n* = 34	
Postoperative shivering	4 (12.5%)	12 (35.3%)	0.031
Recovery time in PACU (min)	46 (41–49)	50 (44–54)	0.038
Hospital stay after surgery (day)	2 (2–3)	2 (2–3)	0.425
Clavien-Dindo classification Ⅲ or more	2 (6.3%)	1 (2.9%)	0.608

Data are presented as median (25–75% interquartile range) or *n* (%). PACU, post-anesthetic care unit.

## Data Availability

The datasets generated and/or analyzed during the current study are not publicly available because disclosing patients’ personal information is against the law, but only de-identified datasets are available from the corresponding author upon reasonable request.
